# Banzhilian formula alleviates psoriasis-like lesions *via* the LCN2/MMP-9 axis based on transcriptome analysis

**DOI:** 10.3389/fphar.2023.1055363

**Published:** 2023-03-06

**Authors:** Meng Xing, Xiaoning Yan, Jiangtao Guo, Wenbin Li, ZhangJun Li, Chun Dong, Jiao Guo, Keshen Qu, Ying Luo

**Affiliations:** ^1^ Department of Dermatology, Shaanxi Hospital of Traditional Chinese Medicine, Xi’an, China; ^2^ The Second Affiliated Hospital of Xi’an Jiaotong University, Xi’an, China; ^3^ The Second Clinical Medical College, Shaanxi University of Chinese Medicine, Xianyang, China; ^4^ Department of Dermatology, The Second Affiliated Hospital of Shaanxi University of Chinese Medicine, Xianyang, China; ^5^ Department of Dermatology, Yueyang Hospital of Integrated Traditional Chinese and Western Medicine, Shanghai University of Traditional Chinese Medicine, Shanghai, China; ^6^ Institute of Dermatology, Shanghai Academy of Traditional Chinese Medicine, Shanghai, China

**Keywords:** psoriasis, Banzhilian formula, Chinese medicine, transcriptomic analysis, RNA sequencing analysis

## Abstract

**Introduction:** Oral Banzhilian formula (BZLF) is effective in the clinical treatment of psoriasis. However, the effectiveness and mechanism of different drug delivery routes deserve further study.

**Methods:** First, we established the mouse model of psoriasis using imiquimod (IMQ), and high-performance liquid chromatography (HPLC) was used for the quality control of BZLF. Secondly, Total RNA Sequencing and bioinformatics analysis were used to explore the regulatory mechanism of BZLF in improving psoriatic lesions. Finally, further verification was based on animal experiments.

**Results:** we externally applied BZLF for skin lesions in an imiquimod-induced psoriasis mouse model and found that BZLF alleviated psoriasis-like skin lesions while inhibiting the expression of Ki67 and inflammatory factors (*Il17a*, *Tnf-α*, *S100a7* and *Cxcl1*) in skin lesions. Transcriptome sequencing results suggested that BZLF inhibited signalling pathways closely related to psoriatic inflammation, such as the IL-17 signalling pathway, chemokine signalling pathway, TNF signalling pathway, and NF-kappa B signalling pathway, and the protein-protein interaction (PPI) network identified LCN2 as one of the core target genes and screened out its regulated downstream gene MMP9.

**Discussion:** Our findings suggest that the anti-psoriatic mechanism of BZLF involved in downregulating the LCN2/MMP-9 axis.

## 1 Introduction

Psoriasis is a chronic inflammatory skin disease with a long course and a tendency to recur. Its clinical manifestations are mainly erythema and scales on the whole body. The prevalence of psoriasis is approximately 0.51%–11.43% (Michalek et al., 2017). The long course of the disease and the high risk of comorbidities, such as cardiovascular disease, tumors, and metabolic syndrome (Fernández-Armenteros et al., 2018; Lee et al., 2019), seriously affect patient health and quality of life, bringing a heavy burden to the social economy (Thomsen et al., 2019). Although biological agents such as interleukin (IL)-17/IL-23 have made certain achievements in recent years (Blauvelt et al., 2022; Thaçi et al., 2022), the treatment of psoriasis still requires the combination of multiple means, especially the application of external medicines. In the guidelines for psoriasis, external medication can be used for both mild psoriasis and for maintenance treatment of psoriasis alone, which is the cornerstone of psoriasis treatment.

Traditional Chinese medicine (TCM) is an important part of complementary and alternative medicine, plays an important role in psoriatic prevention and treatment, and was highlighted in the 2018 edition of the *Guidelines for the Diagnosis and Treatment of Psoriasis in China* (Chinese Medical Association Dermatology Branch Psoriasis Professional Committee, 2019). TCM holds the viewpoint that the principle of external treatment is consistent with the principle of internal treatment. Therefore, a botanical drug in the clinic can be given both orally and externally or in medicated baths. Banzhilian formula (BZLF) is a common TCM prescription for psoriasis that consists of nine traditional Chinese medicinal materials: *Scutellaria barbata* D. Don, *Dictamnus dasycarpus* Turcz., *Cnidium monnieri* (L.) Cuss., *Saposhnikovia divaricate* (Turcz.) Schischk., Cicadae Periostracum, *Dioscorea collettii* var. *hypoglauca* (Palib.) S. J. Pei and C. T. Ting, *Nepeta cataria* L., *Chrysanthemum indicum* L., and *Taraxacum mongolicum* Hand. -Mazz. Previous clinical studies have confirmed that oral BZLF can effectively improve the PASI score and reduce serum levels of TNF-α and VEGF in patients with psoriasis (Li et al., 2014). Although BZLF has a curative effect on psoriasis without significant side effects, the efficacy and mechanism of its external application to treat psoriasis need to be further studied.

RNA sequencing (RNA-seq) is an important tool for transcriptomic research and provides a new method for multicomponent and multitarget research in botanical drugs. Therefore, we used RNA-seq to study the potential mechanism by which BZLF protects against psoriasis by externally applying BZLF to mice with imiquimod (IMQ)-induced psoriasis to provide a more scientific basis for the subsequent development of external medicines for psoriasis.

## 2 Materials and methods

### 2.1 Pharmaceutical composition and plant material of BZLF

The BZLF consists of nine traditional Chinese medicinal materials: *Scutellaria barbata* D. Don [Lamiaceae; *Scutellaria barbata*], *Dictamnus dasycarpus* Turcz. [Rutaceae; Cortex Dictamni], *Cnidium monnieri* (L.) Cusson [Umbelliferae; Fructus Cnidii], *Saposhnikovia divaricata* (Turcz.) Schischk. [Umbelliferae; Saposhnikoviae Radix], Cicadae Periostracum [Cryptotympana pustulata Fabricius; Cicada Slough], *Dioscorea collettii* var. *hypoglauca* (Palib.) S. J. Pei and C. T. Ting [Dioscoreaceae; Dioscorea hypoglauca Palibin], *Nepeta cataria* L. [Lamiaceae; Schizonepeta], *Chrysanthemum indicum* L. [Compositae; wild chrysanthemum], *Taraxacum mongolicum* Hand. -Mazz. [Asteraceae; dandelion]. The ratio is 6:10:5:5:5:5:5:5:5. All materials were extracted, concentrated, dried, and processed into granules. The granules of each material were purchased from Yueyang Hospital of Integrated Traditional Chinese and Western Medicine, Shanghai University of Traditional Chinese Medicine. The granules used in the current study were provided by Sichuan Neo-Green Pharmaceutical Technology Development Co., Ltd. (Sichuan, China).

### 2.2 HPLC

HPLC used a Welch Ultimate PLUS C18 250 × 4.6 mm, 5 μm column; a DAD detector with a 220 nm detection wavelength; a flow rate of 1 mL/min; a sample size of 10 μL at 30 °C. Mobile phase A was 0.2% phosphoric acid aqueous solution; and mobile phase B was 0.1% trifluoroacetic acid acetonitrile. The standard application was as follows: caffeic acid (main active ingredient of *Taraxacum mongolicum Hand*.-Mazz*.*, CAS No. 331-39-5, purity ≥98%), baicalin (main active ingredient of *Scutellaria barbata* D. Don, CAS No. 27740-01-8, purity ≥98%), obakunone (main active ingredient of *Dictamnus dasycarpus* Turcz., CAS No. 751-03-1, purity ≥98%), fraxinellone (main active ingredient of *Dictamnus dasycarpus* Turcz., CAS No. 28808-62-0, purity ≥98%), and osthole (main active ingredient of *Cnidium monnieri* (L.) Cusson, CAS No. 484-12-8, purity ≥98%).

### 2.3 Animals

Male specific-pathogen-free (SPF)-grade BALB/c mice (20–25 g body weight, 5–6 weeks old) were provided by the Shanghai Medical Experimental Animal Center (SCXK Shanghai 2013-0016, Shanghai, China). Mice were maintained in a controlled environment with room temperature at 22–23°C and a 12 h dark/light cycle. The fodder (Shanghai Pu Lu Tong Biological Technology Co., Ltd.) and sterile water were applied. All procedures were approved by and carried out in accordance with regulations of the Ethics Committee of Yueyang Hospital affiliated with the Shanghai University of Traditional Chinese Medicine (No. YYLAC-2021-107).

### 2.4 Experimental grouping and model establishment

Mice were randomly divided into three groups after shaving their back hair (2 × 2 cm^2^). The mice psoriasis model was induced externally by imiquimod (IMQ) (Sichuan Mingxin Pharmaceutical Co., Ltd., Sichuan, China, Drug approval No. H20030128), and the control group was coated with petroleum jelly (Nanchang Baiyun Pharmaceutical Co., Ltd., Jiangxi, China, Drug Approval No. F20050006). The BZLF treatment was used as an external application at 20.4, 40.8, and 81.6 mg/cm^2^/day dosage. The groups were treated as follows: 1) control group: back was treated with 62.5 mg petroleum jelly. 2) IMQ + NS group: back was treated with 62.5 mg IMQ cream for 6 h, then externally covered with a 0.9% NaCl solution and fixed with a layer of gauze and medical polyurethane film. 3) IMQ + BZLF group: back was treated with 62.5 mg IMQ cream for 6 h, then externally covered with 20.4/40.8/81.6 mg/cm^2^ BZLF, respectively, and fixed with a layer of gauze and medical polyurethane film. All treatments were performed by applying the IMQ cream on day 0 and then once daily for 10 consecutive days. PASI scores were used to determine the severity of skin inflammation on the backs of the mice. On day 10, the mice were euthanized by inhalation of carbon dioxide. The lesions on the backs of the mice were collected as reserves.

### 2.5 Total RNA Sequencing

Total RNA was extracted using the mirVana miRNA Isolation Kit (Ambion) following the manufacturer’s protocol. RNA integrity was evaluated using the Agilent 2100 Bioanalyzer (Agilent Technologies, Santa Clara, CA, United States). The samples with RNA integrity number (RIN) ≥ 7 were subjected to the subsequent analysis. The libraries were constructed using TruSeq Stranded Total RNA with Ribo-Zero Gold according to the manufacturer’s instructions. Then, these libraries were sequenced on the Illumina sequencing platform (HiSeqTM 2500), and 150 bp/125 bp paired-end reads were generated.

### 2.6 mRNA quantitative and differential analysis

Aligning the sequencing reads of each sample with the sequence of mRNA transcript sequences, known lncRNA sequences and lncRNA prediction sequences by Bowtie2, and using eXpress for quantitative gene analysis, the FPKM values and counts values (the number of reads for each gene in each sample) were obtained. The estimateSizeFactors function of the DESeq2R package was used to normalize the counts, and the nbinomTest function was used to calculate *p*-value and foldchange values for the difference comparison. Differential transcripts with *p*-values <0.05 and foldchange >2 were selected.

### 2.7 Gene Ontology (GO) and Kyoto Encyclopedia of Genes and Genomes (KEGG) analysis

The GO and KEGG pathway databases were used to analyze the functional interpretation and KEGG pathway of genes (Huang et al., 2009). The numbers of genes were counted according to GO terms and KEGG pathways. Fisher’s exact test was used to obtain the *p*-value, and multiple hypothesis testing was performed to obtain the *q*-value. The significantly enriched GO terms were defined as *p*-value <0.05. The same analytic approach was performed to identify significantly enriched KEGG terms of genes.

### 2.8 Construction of core targets for treatment of psoriasis-like lesions with BZLF

The STRING database (https://string-db.org) was used to construct an active ingredient–target and target interaction network, while Cytoscape 3.8 software was used for visualization and network topology analysis. Using the average of degree centrality (DC), betweenness centrality (BC), and closeness centrality (CC) as the card values, the nodes meeting the three card values were selected as the key nodes of BZLF potential targets.

### 2.9 H&E solution and immunohistochemistry (IHC)

On day 10, the mice were euthanized by inhaling carbon dioxide. The central lesions were fixed with 4% formalin solution, dehydrated, embedded in paraffin, sectioned, and stained with H&E solution, and IHC was performed. Anti-Ki-67 antibody (1:50, ab16667, Abcam) was used in IHC. Quantitative methods for determining the epidermal thickness and positive cell rate have been described in previous studies and were the same as our previous study (Kuai et al., 2021).

### 2.10 RT-PCR

The lesions were collected for RT-PCR on day 10. The experimental process included total RNA extraction, synthesis of cDNA from RNA by reverse transcriptase, and amplification and synthesis of target fragments using cDNA as a template under the action of DNA polymerase. The specific process and data statistical methods were the same as in our previous study (Kuai et al., 2018). Primer sequences are shown in Supplementary Table S1.

### 2.11 Western blot

Briefly, after the samples were removed from the back of the mice, the total protein was extracted. Then, the samples were loaded for electrophoresis, followed by membrane transfer, sealing, and antibody incubation and detection. The antibodies for Western blotting included anti-LCN2 antibodies (26991-1-AP; Proteintech), anti-MMP9 antibodies (ab228402; Abcam), and β-actin (ab8226; Abcam).

### 2.12 Biochemical indicators

On day 10, the mice were euthanized by inhaling carbon dioxide. Blood was collected to separate serum for detection of ALT, AST and CRE using a BECKMAN LX20 full automatic biochemical analyzer.

### 2.13 Statistical methods

Data were analyzed *via* SPSS 24.0 (IBM, New York, United States) and described as mean ± standard deviation. The two groups were compared using the *t*-test. *p* < 0.05 was set as statistically significant.

## 3 Results

### 3.1 Quality control of BZLF

**FIGURE 1:**
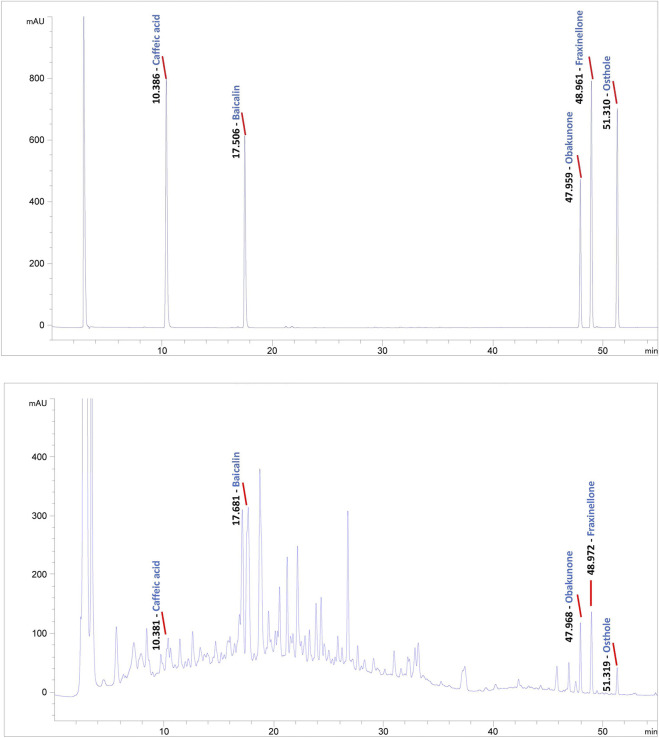
Quality control of Banzhilian formula (BZLF) through caffeic acid, baicalin, obakunone, fraxinellone, and osthole. Caffeic acid, baicalin, obakunone, fraxinellone, and osthole were detected in both the positive control and the BZLF samples, but neither was found in the negative control.

To conduct quality control of BZLF, we selected five representative main ingredients in BZLF for analysis. Caffeic acid, baicalin, and obakunone are the main active ingredients in *Taraxacum mongolicum* Hand. -Mazz., *Scutellaria barbata* D. Don, and *Dictamnus dasycarpus* Turcz., respectively, and all three ingredients have been reported to have some palliative effects on psoriasis (Yang et al., 2018; Wu L. et al., 2020; Wu X. et al., 2020; Dimitris et al., 2020). Furthermore, fraxinellone, a main active ingredient in *Dictamnus dasycarpus* Turcz., shows potent anti-inflammatory and immunomodulatory effects that are hepatoprotective and can treat proliferative diseases (Bailly and Vergoten, 2020). Additionally, osthole, a principal component of *Cnidium monnieri* (L.) Cusson, exerted inhibitory effects on hypoxic HCT116 cells that may be associated with eukaryotic initiation factor 2 alpha phosphorylation-mediated apoptosis and the translational repression of hypoxia-inducible factor-1 (HIF-1) (Peng and Chou, 2022). We previously confirmed that HIF-1α was highly expressed in psoriatic lesions and could be affected by pathways regulated by TCM (Qu et al., 2021). Collectively, we chose caffeic acid, baicalin, obakunone, fraxinellone, and osthole to preliminarily establish quality control for BZLF (Figure 1).

### 3.2 BZLF relieved skin inflammation and keratinocyte (KC) proliferation in mice with IMQ-induced psoriasis

**FIGURE 2:**
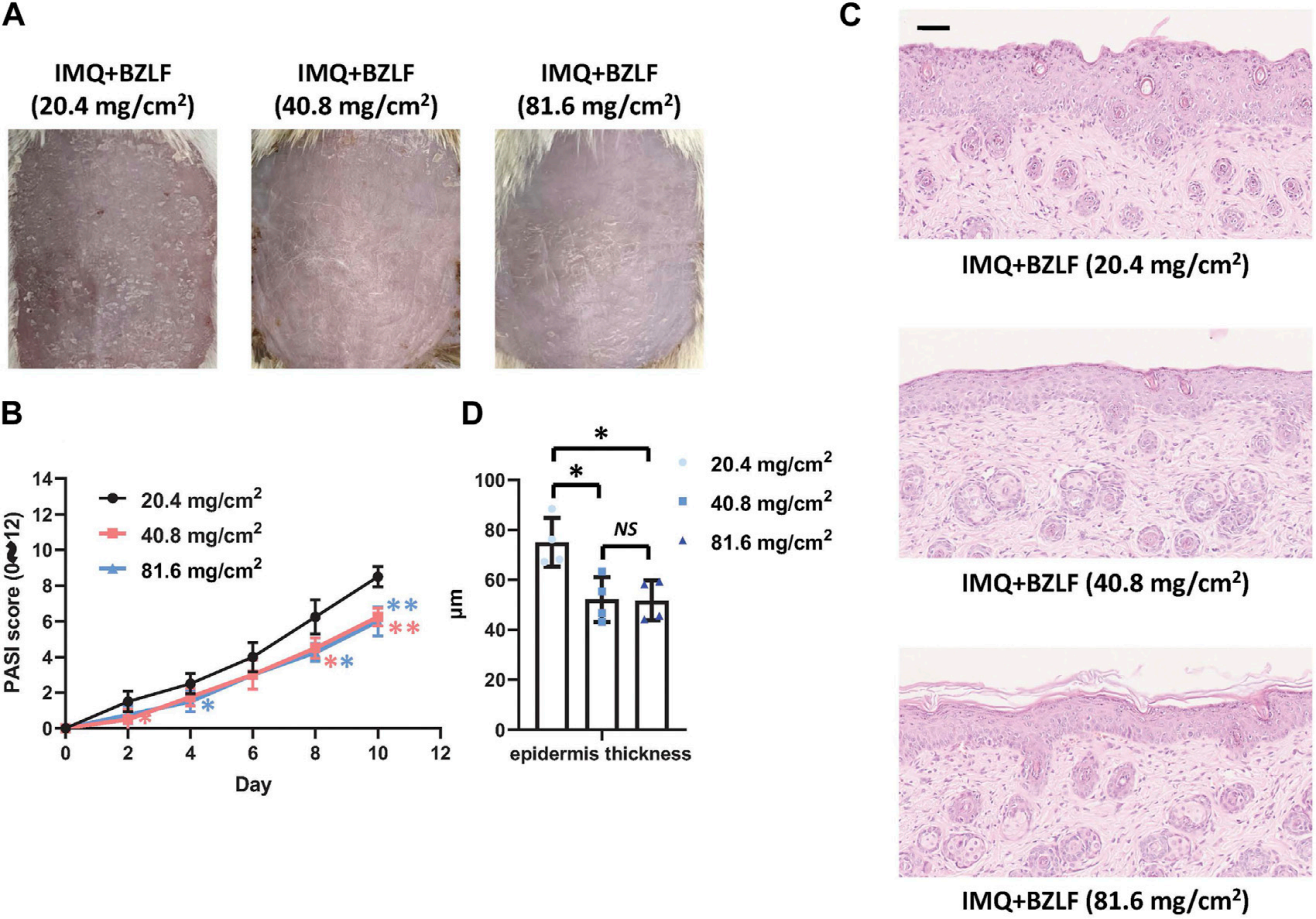
Effect of different doses of Banzhilian formula (BZLF) on IMQ-induced psoriasis-like skin lesions. **(A)** Appearance of back lesions in each group on day 10. **(B)** Psoriasis area severity index (PASI) score (0–12). **(C)** Representative H&E sections of skin lesions on the 10th day (×200). **(D)** Quantification of epidermal thickness in the back lesions. Scale bar: 100 µm. The data are expressed as mean ± SD. Four skin lesions in each group were included for analysis. *p < 0.05, **p < 0.01, compared with the 20.4 mg/cm^2^ group. ns, not significant; 40.8 mg/cm^2^ group compared with the 81.6 mg/cm^2^ group.

**FIGURE 3:**
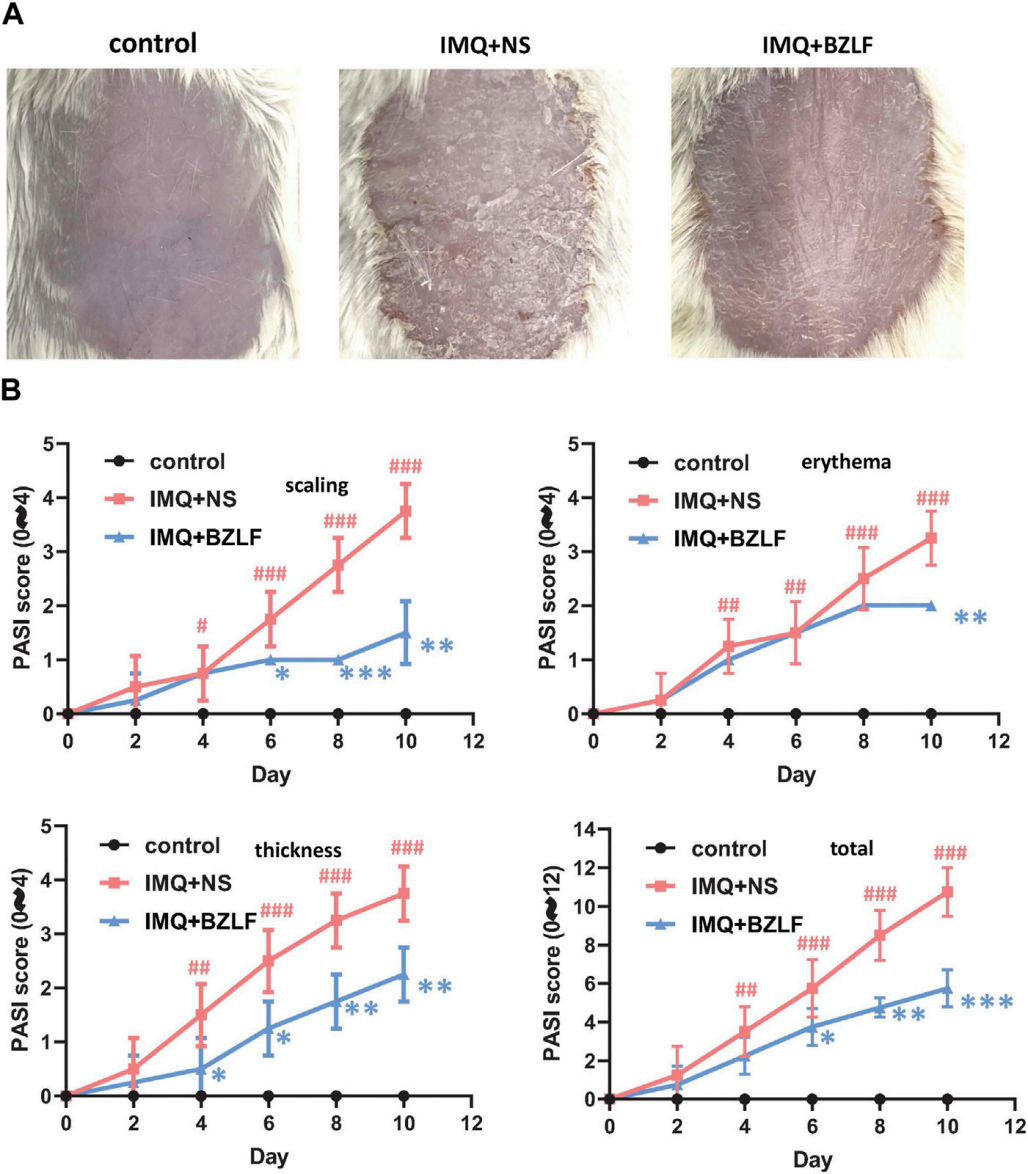
Banzhilian formula (BZLF) alleviates IMQ-induced psoriasis-like skin lesions in mice. **(A)** Appearance of the back lesions of the mice in each group on the 10th day. **(B)** Psoriasis area severity index (PASI) score (0–4) with scales, thickness, erythema, and total score. The data are expressed as mean ± SD. Four skin lesions from each group were included for analysis. ^#^ *p* < 0.05, ^##^ *p* < 0.01, ^###^ *p* < 0.001, compared with the control group. *p < 0.05, **p < 0.01, ***p < 0.001, compared with the IMQ + NS group.

**FIGURE 4:**
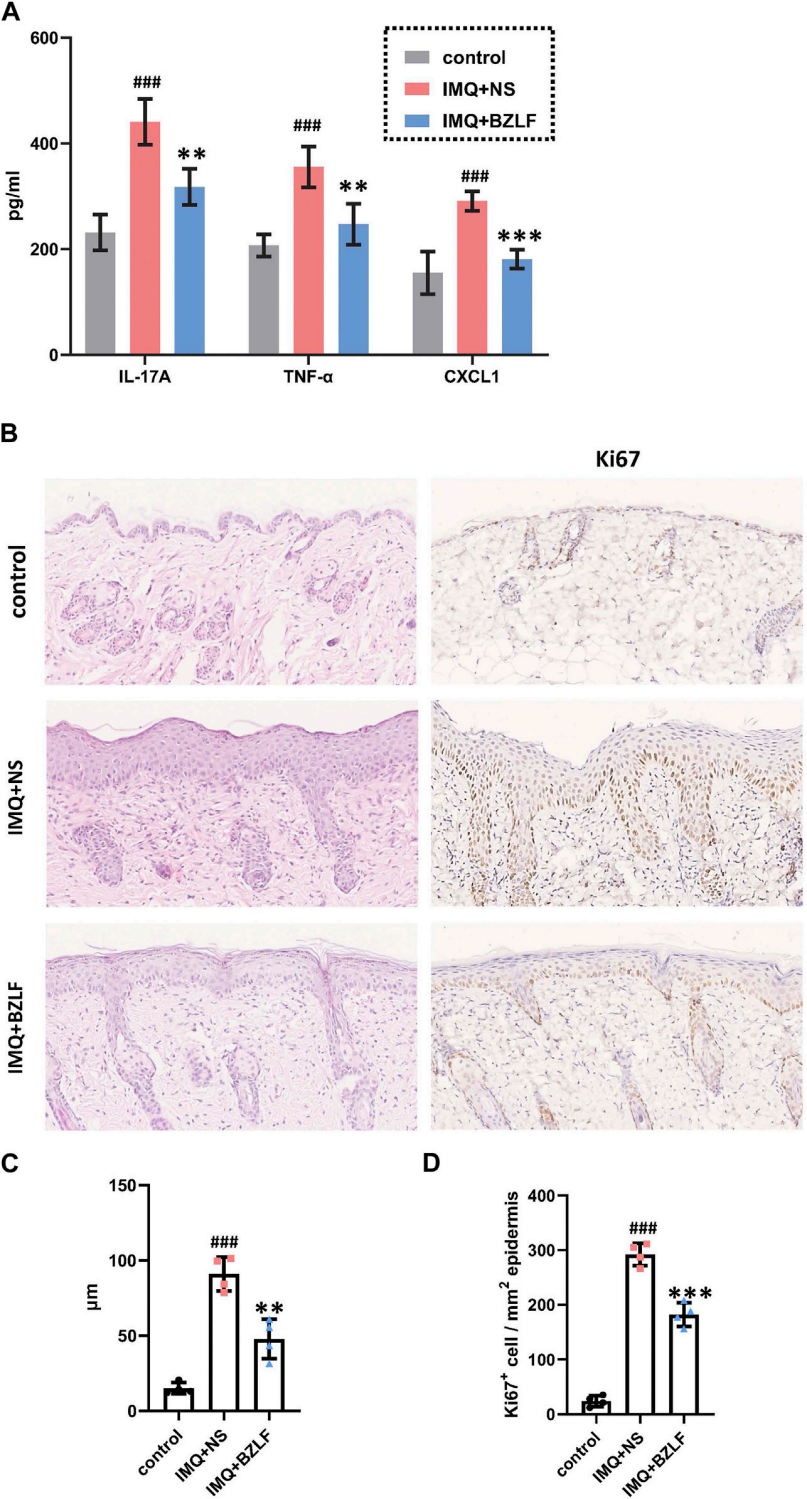
Banzhilian formula (BZLF) inhibits inflammation and epidermal proliferation in IMQ-induced psoriasis-like lesions. **(A)** mRNA expression of IL-17A, TNF-α, and CXCL1 in skin lesions of each group on the 10th day. **(B)** Representative H&E sections of skin lesions on the 10th day (×200) (left). Representative immunohistochemistry sections of Ki67 nuclear staining (brown) of the back lesions (×200) (right). **(C)** Quantification of epidermis thickness in back lesions. **(D)** Quantification of Ki67^+^ cells in skin lesions. Scale bar: 100 µm. The data are expressed as mean ± SD. Four skin lesions from each group were included for analysis. ^#^ *p* < 0.05, ^##^ *p* < 0.01, ^###^ *p* < 0.001, compared with the control group. *p < 0.05, **p < 0.01, ***p < 0.001, compared with the IMQ + NS group.

To elucidate the effect of the external application of BZLF on psoriasis, we initially established a psoriasis mouse model with IMQ and determined that 40.8 mg/cm^2^ was the most effective dose of BZLF (Figure 2). The results showed that on the 10th day of BZLF treatment, the symptoms of psoriasis-like erythema and scaling were significantly improved and were accompanied by a significant decrease in the PASI score (Figures 3A, B). Next, to examine the influence of skin inflammation levels, we measured the expression of inflammatory cytokines related to psoriasis and confirmed that *Il17a*, *Tnf-α*, and *Cxcl1* mRNA expression in IMQ-induced lesions decreased after treatment with BZLF (Figure 4A). Histopathology suggested that BZLF treatment could reduce epidermal thickness (Figures 4B, C) in IMQ-induced lesions. Moreover, excessive KC proliferation was prevented by BZLF (Figures 4B, D). On the other hand, we examined changes in body weight and the potential toxicity of BZLF in mice after the external application of BZLF and found that it had no significant effect on the body weight of psoriatic mice (Supplementary Figure S1A). Security index tests showed that BZLF did not influence liver or kidney function (Supplementary Figure S1B). In conclusion, we showed that external application of BZLF could alleviate psoriasis-like skin lesions with high safety.

### 3.3 RNA-seq analysis of BZLF-regulated genes in IMQ-induced psoriasis-like lesions

#### 3.3.1 Differentially expressed genes (DEGs) following BZLF treatment

To further examine the mechanism by which external application of BZLF can treat psoriasis, RNA-seq was performed on the control group and IMQ-induced lesions on day 10 of BZLF or NS treatment. A total of 86.53 G of clean data were obtained. The effective data amount in each sample ranged from 6.98 to 7.42 G, Q30 basic groups ranged from 94.79% to 98.18%, and the average GC content was 51.598%. The FPKM values and the principal component analysis (PCA) are shown in Supplementary Figure S2.

**FIGURE 5:**
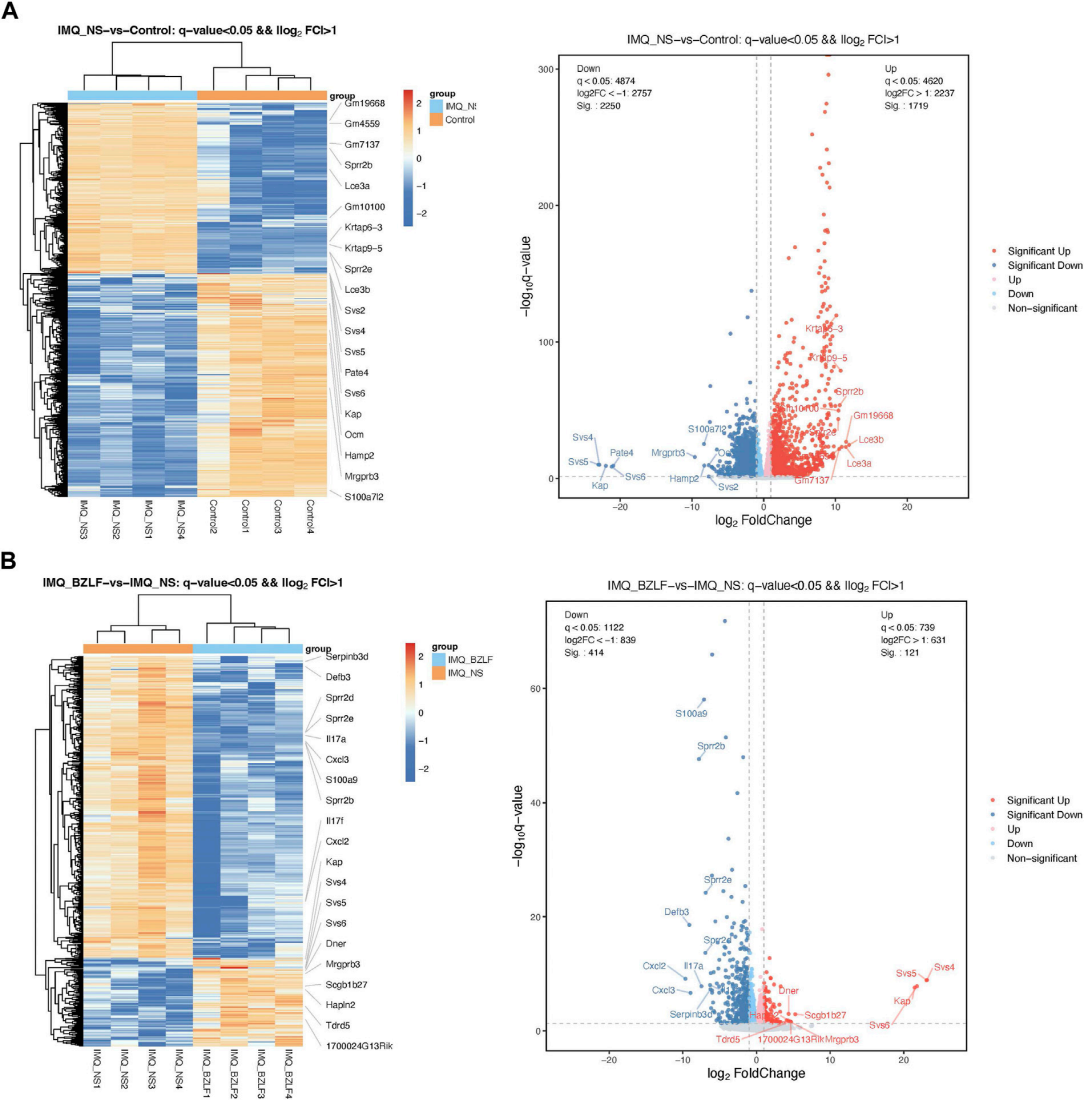
Differentially expressed genes (DEGs) in the different groups. Cluster analysis of DEGs among samples and groups. The color of the heat map indicates the relative gene expression. The deeper orange color indicates higher gene expression, whereas the deeper blue color indicates lower gene expression (Left). Differential expression volcano map reflecting the differently expressed genes. Gray indicates genes with no significant difference, red indicates genes that are significantly upregulated, and blue indicates genes that are significantly downregulated (Right). **(A)** IMQ + NS group compared with the control group. **(B)** BZLF group compared with the IMQ + NS group.

**FIGURE 6:**
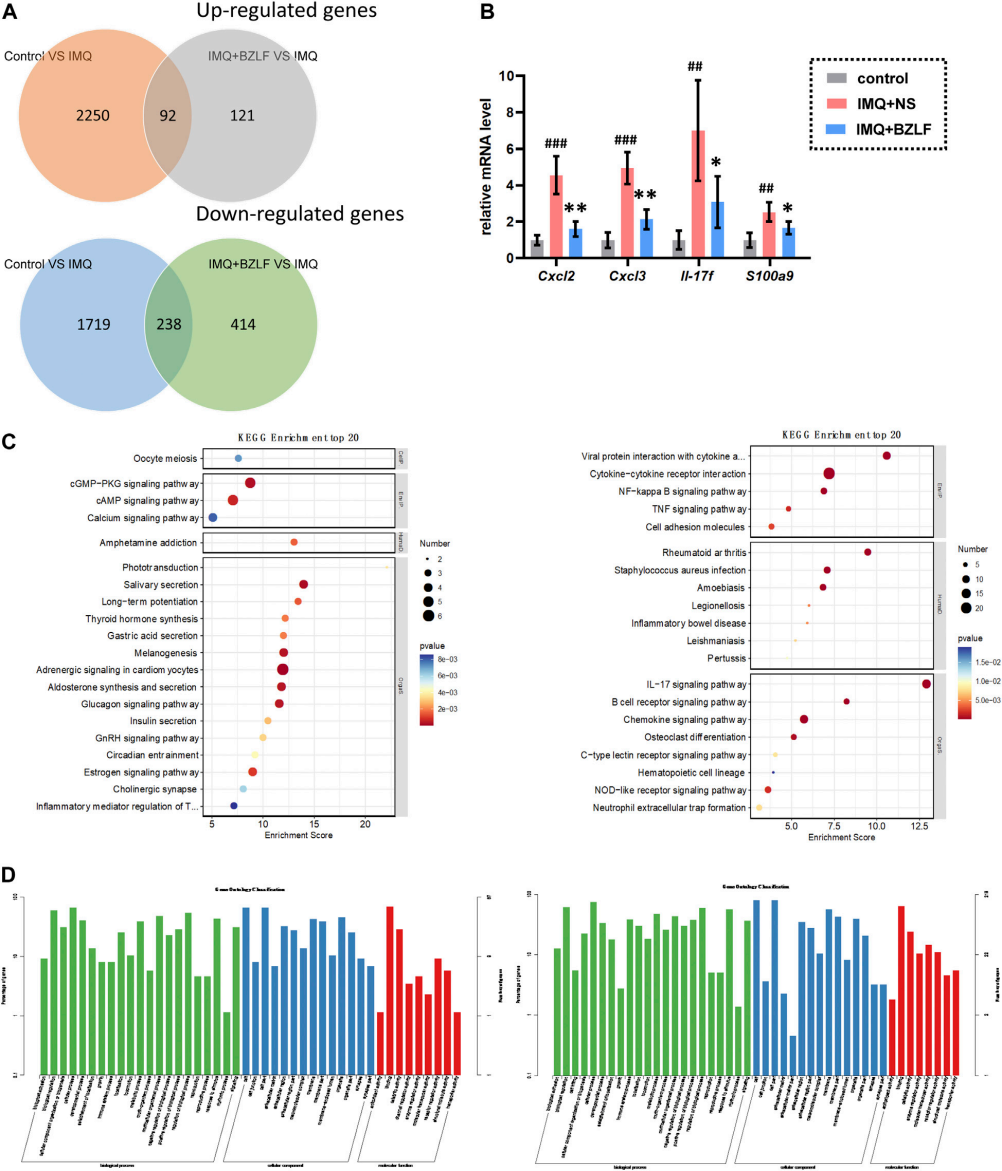
Experimental verification and enrichment analysis of differentially expressed genes (DEGs) after treatment with Banzhilian formula (BZLF). (**A)** Venn diagram of up- and downregulated differentially expressed genes in the BZLF vs. the IMQ + NS group and the control group vs. the IMQ + NS group. **(B)** RT-PCR showed the mRNA expressions of the top four DEGs. **(C)** Enriched KEGG analysis of up- (left) and downregulated (right) DEGs. **(D)** Enriched Gene Ontology analysis of up- (left) and downregulated(right) DEGs. ^#^ *p* < 0.05, ^##^ *p* < 0.01, ^###^ *p* < 0.001, compared with the control group. *p < 0.05, **p < 0.01, ***p < 0.001, compared with the IMQ + NS group.

DESeq2 software was used to standardize the counts of each sample gene. Then, the DEGs were screened according to *p*-values <0.05 and foldchange >2. Between normal skin and psoriasis-like lesions, a total of 3,969 DEGs were identified, of which 1,719 were upregulated, and 2,250 were downregulated (Figure 5A). In comparing untreated psoriasis-like lesions versus lesions treated with BZLF, there were 535 DEGs, of which 121 were upregulated genes and 414 were downregulated genes (Figure 5B). To further identify the DEGs related to psoriasis following BZLF treatment, we examined the intersection of the upregulated and downregulated genes. Finally, 330 overlapping DEGs closely related to psoriasis were screened out, including 92 upregulated genes and 238 downregulated genes (Figure 6A). Next, we verified the top four DEGs (Figure 6B).

#### 3.3.2 Biological characteristics of the potential pathways associated with BZLF treatment

To further investigate the potential pathways associated with external BZLF treatment for psoriasis, we evaluated the screened DEGs by KEGG and GO analyses. KEGG analysis indicated that externally applied BZLF upregulated pathways that included the calcium signaling pathway, the cAMP signaling pathway, the cGMP−PKG signaling pathway, aldosterone synthesis and secretion, and salivary secretion (Figure 6C). GO analysis showed that enzyme regulator activity, calcium ion binding, heparin binding, and the neurotransmitter catabolic process were the most significantly upregulated gene categories (Figure 6D). In addition, the IL-17 signaling pathway, cytokine−cytokine receptor interaction, the chemokine signaling pathway, the NOD-like receptor signaling pathway, the TNF signaling pathway, the NF-kappa B signaling pathway, and cell adhesion molecules were downregulated following the external application of BZLF, as shown by KEGG analysis (Figure 6C). The cysteine-type endopeptidase inhibitor, cytokine activity, chemokine activity, cornified envelope, inflammatory response, neutrophil chemotaxis, and immune system process were the most significantly downregulated gene categories revealed by GO analysis (Figure 6D).

### 3.4 Core targets of BZLF in IMQ-induced psoriasis-like lesions

Next, we constructed the core targets based on screened DEGs by RNA-seq. After excluding targets with confidences less than 0.4 in the STRING database, we imported the interaction information file containing 167 target proteins into Cytoscape to construct the protein‒protein interaction (PPI) network. By calculating the topological value, 26 core targets were screened out according to the following criteria: DC ≥ 8.31, BC ≥ 0.017, and CC ≥ 0.278 (Supplementary Table S2). These core targets are represented by red and blue nodes in the PPI network, respectively; the red nodes represent the top 10 core targets (Il1b, Itgam, Il17a, CCL20, PTGS2, CXCL2, SELL, MPO, TREM1, and LCN2) that were screened based on DC values (Supplementary Figure S3).

### 3.5 BZLF downregulates lipocalin-2 (LCN2) expression in IMQ-induced psoriasis-like lesions

**FIGURE 7:**
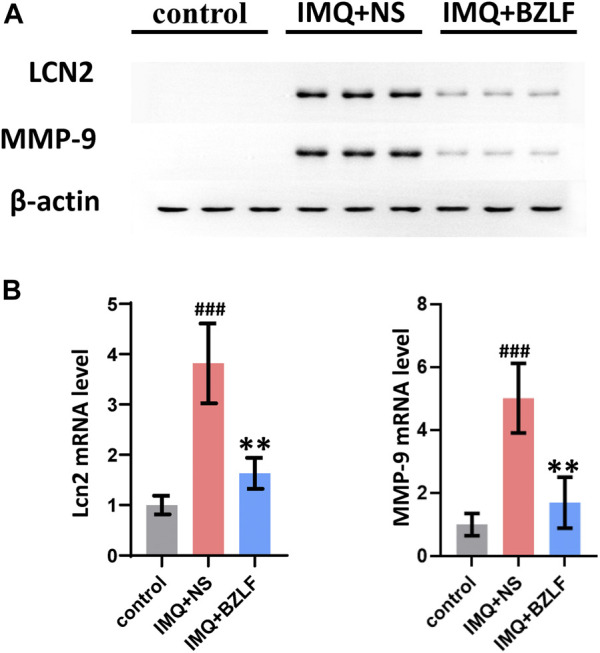
Banzhilian formula (BZLF) downregulates LCN2 and MMP-9 expression in lesions. Expression of LCN2 and MMP-9 in skin lesions assessed by Western blot **(A)** and RT-PCR **(B)** on day 10. The data are expressed as mean ± SD. Three skin lesions in each group were included for analysis. *p < 0.05, **p < 0.01, ***p < 0.001, compared with the IMQ + NS group.

LCN2 is one of the top 10 core targets in the PPI network and likely mediates biological processes involved in the external application of BZLF for treating psoriasis. LCN2, which is a member of the Lipocalin superfamily, is a 25-kDa secreted protein that is expressed in a variety of cells and is involved in the transport of lipophilic small molecules such as steroids, lipopolysaccharides, iron, and fatty acids. Recent studies have suggested that LCN2 inhibits NLRC4 signaling through SREBP2 to alleviate psoriatic dermatitis (Ma et al., 2022). Therefore, we measured the mRNA and protein expression of LCN2 in the different groups on the 10th day and found that the mRNA and protein expression were upregulated in IMQ-induced psoriasis-like skin lesions compared with those in the control group, whereas BZLF inhibited LCN2 mRNA and protein expression (Figures 7A, B).

### 3.6 BZLF alleviates IMQ-induced psoriasis-like lesions by inhibiting the LCN2/MMP-9 axis

Although we confirmed that externally applied BZLF ameliorates psoriasis-like lesions by inhibiting LCN2, we found that its downstream genes *Serbp2* and *Nlrc4* were not in our DEG list, as determined by RNA-seq. To further investigate the mechanism by which BZLF regulates LCN2, we conducted a protein‒protein interaction analysis using the Search Tool for the Retrieval of Interaction Gene/Proteins (STRING) database based on our sequencing results (Supplementary Table S3). The results showed that among the DEGs, MMP-9, LRP2, TIMP1, IL17A, and SAA1 had higher combined scores for LCN2, and the MMP-9 combined scores were the highest. Therefore, MMP-9 was chosen to examine the possible mechanism by which BZLF downregulates LCN2 to improve psoriasis-like skin lesions. Western blot (WB) showed that BZLF significantly reduced the protein expression of MMP-9 (Figure 7A), while RT-PCR indicated the same change at the mRNA level (Figure 7B).

## 4 Discussion

In this study, we administered BZLF orally and found that external application of BZLF could improve erythema and scaling and reduce epidermal thickness in IMQ-induced psoriasis-like mouse skin lesions. According to the two main pathological characteristics of inflammatory cell infiltration and abnormal keratinocyte proliferation in psoriasis, we confirmed that BZLF downregulated the expression of inflammatory factors (*Il17a*, *Tnf-α*, and *Cxcl1*) in psoriasis-like lesions and inhibited the expression of the keratinocyte proliferation marker Ki67. Therefore, we believe that the external application of BZLF has a potential therapeutic effect and research value in treating psoriasis.

Given the complexity of the composition and mechanism of action of traditional Chinese medicinal materials, we tried to elucidate the pharmacological mechanism of the external application of BZLF from a holistic perspective. We used RNA-seq to analyze skin tissue samples in the different groups, took the intersection of DEGs in the different groups, and verified the accuracy of the sequencing results by RT-PCR.

The therapeutic effects of BZLF were regulated by core genes, especially *Lcn2*, which we identified through bioinformatics analysis of the RNA-seq results. We further used Western blotting and RT-PCR to confirm that BZLF downregulated the expression of LCN2 in IMQ-induced psoriasis-like mouse skin lesions. LCN2 is highly expressed in the skin lesions and the serum of patients with psoriasis (El-Hadidi et al., 2014; Wang et al., 2019) and can inhibit the synthesis of keratin, involucrin, and loricrin in KCs, leading to epidermal parakeratosis *via* the Tcf7L1-lipocalin 2 signaling axis. LCN2 can also recruit inflammatory cells, such as T cells and neutrophils, to skin lesions through the IL-23/IL17, p38-MAPK, and ERK-1/2 signaling pathways (Shao et al., 2016). In addition, LCN2 and other cytokines, such as IL-17, have a synergistic effect on skin cells (Hau et al., 2016). According to these results, we hypothesized that BZLF alleviated inflammation in skin lesions and inhibited the proliferation of KCs, which was associated with the downregulation of LCN2 expression.

To further examine the regulatory mechanism by which BZLF acts on LCN2 to improve psoriasis-like skin lesions, we used the STRING database to predict target genes that might interact with LCN2 based on the DEGs from the RNA-seq results. The analysis results showed that MMP-9, which is an inflammatory factor, had the highest combined scores. Next, we found that MMP-9 was significantly elevated in the IMQ-induced psoriasis mouse model, and BZLF downregulated its expression at both the protein and RNA levels. Neutrophil infiltration and tortuous telangiectasia are pathological features of psoriasis (Chau et al., 2017), and MMP-9 can decompose a 62-amino acid peptide from IL-8 (CXCL8/CL8) to increase the chemotactic activity of neutrophils (Mieke et al., 2018). MMP-9 also participates in angiogenesis by releasing vascular endothelial growth factor (VEGF) (Zeng et al., 2020). Moreover, studies have indicated that MMP-9 induces skin vasodilation and hyperpermeability by activating vascular endothelial cells in skin, thereby promoting the development of psoriatic lesions (Chen et al., 2020). At present, MMP-9 has been shown to play an important role in the pathogenesis of psoriasis (Kvist-Hansen et al., 2021; Lu et al., 2022). Therefore, we hypothesized that BZLF alleviated inflammation in psoriasis-like skin lesions and inhibited the proliferation of KCs, which was related to the downregulation of the LCN2/MMP-9 axis.

## 5 Conclusion

We examined the efficacy of the external application of BZLF in the treatment of psoriasis and analyzed its mechanism of action by RNA-seq and experimental validation. The specific regulatory mechanism of BZLF mainly involves the upregulation of lipid metabolism-related signaling pathways, downregulation of inflammation-related signaling pathways, and inhibition of the LCN2/MMP-9 axis. This study conducted a preliminary exploration of the external application of BZLF and provided an important material basis for the subsequent research and development of external TCM treatments.

## Data Availability

The data generated from this article can be found in the Gene Expression Omnibus database (https://www.ncbi.nlm.nih.gov/geo/), using accession number GSE223468.
