# Supplementary material for: Banzhilian formula alleviates psoriasis-like lesions via the LCN2/MMP-9 axis based on transcriptome analysis
Source: Front Pharmacol. 2023 Mar 6;14:1055363. doi: 10.3389/fphar.2023.1055363 (PMC10025347; doi:10.3389/fphar.2023.1055363)
Supplement: Supplementary file 1 [file Table1.DOCX]

**Table S1 Primer sequences for RT-PCR**

| GENE NAME | F | R |
| --- | --- | --- |
| CXCL2 | CCAACCACCAGGCTACAGG | GCGTCACACTCAAGCTCTG |
| LCN2 | GGCCAGTTCACTCTGGGAAA | TGGCGAACTGGTTGTAGTCC |
| MMP9 | TGGTCTTCCCCAAAGACCTG | CACAGCGTGGTGTTCGAATG |
| CXCL3 | GAAAGGAGGAAGCCCCTCAC | TGGCCAGCCAAGGAATACTG |
| CXCL1 | ACTCAAGAATGGTCGCGAGG | GTGCCATCAGAGCAGTCTGT |
| TNF-α | GGACTAGCCAGGAGGGAGAA | CGCGGATCATGCTTTCTGTG |
| IL-17f | TGCTACTGTTGATGTTGGGAC | CAGAAATGCCCTGGTTTTGGT |
| IL-17A | GCTGACCCCTAAGAAACCCC | GAAGCAGTTTGGGACCCCTT |
| S100a9 | CCAACATCTGTGACTCTTTAGCC | GCTCAGCTGATTGTCCTGGT |

**Table S2** **Core targets**

| Name | Degree | Betweenness Centrality | Closeness Centrality |
| --- | --- | --- | --- |
| IL1B | 52 | 0.27615383 | 0.43799472 |
| ITGAM | 45 | 0.11547751 | 0.41191067 |
| IL17A | 38 | 0.03043512 | 0.38248848 |
| CCL20 | 30 | 0.08163339 | 0.38248848 |
| PTGS2 | 27 | 0.14711819 | 0.4 |
| CXCL2 | 27 | 0.02384072 | 0.37471783 |
| SELL | 26 | 0.0376532 | 0.35546039 |
| MPO | 26 | 0.02424064 | 0.37387387 |
| TREM1 | 24 | 0.02933765 | 0.35622318 |
| LCN2 | 22 | 0.13116572 | 0.31619048 |
| SPRR1B | 21 | 0.03359795 | 0.36403509 |
| TLR1 | 19 | 0.02451712 | 0.3487395 |
| S100A8 | 17 | 0.12708705 | 0.38694639 |
| SPRR2D | 16 | 0.02772749 | 0.29020979 |
| LCE3D | 15 | 0.04909735 | 0.29590018 |
| IL36G | 14 | 0.05442934 | 0.35394456 |
| FGR | 14 | 0.01741578 | 0.35021097 |
| LTF | 13 | 0.03449498 | 0.33808554 |
| IL36RN | 13 | 0.02914056 | 0.35622318 |
| DEFB4A | 13 | 0.02162945 | 0.34800839 |
| MMP13 | 12 | 0.02072747 | 0.33333333 |
| LCE3E | 12 | 0.01869177 | 0.29225352 |
| CD22 | 11 | 0.04101338 | 0.32046332 |
| CD300LF | 11 | 0.01790967 | 0.32108317 |
| SLPI | 9 | 0.04344659 | 0.35169492 |
| CD79B | 9 | 0.02301223 | 0.29328622 |

**Table S3 Genes of combined-score with LCN2**

| Name | Gene definition | Combined score |
| --- | --- | --- |
| MMP-9 | Matrix metalloproteinase-9 | 0.999 |
| SLC22A17 | Solute carrier family 22 member 17; | 0.998 |
| LRP2 | Low-density lipoprotein receptor-related protein 2; | 0.987 |
| MMP-2 | Matrix metalloproteinase-2 (gelatinase a) | 0.973 |
| MC4R | Melanocortin receptor 4 | 0.973 |
| IL6 | Interleukin-6; | 0.967 |
| TIMP1 | Metalloproteinase inhibitor 1 | 0.966 |
| IL17A | Interleukin-17A | 0.958 |
| SAA1 | Serum amyloid A-1 protein; | 0.956 |
| IL-10 | Interleukin-10; | 0.954 |
