# Supplementary figures and images for: Banzhilian formula alleviates psoriasis-like lesions via the LCN2/MMP-9 axis based on transcriptome analysis
Source: Front Pharmacol. 2023 Mar 6;14:1055363. doi: 10.3389/fphar.2023.1055363 (PMC10025347; doi:10.3389/fphar.2023.1055363)

**A**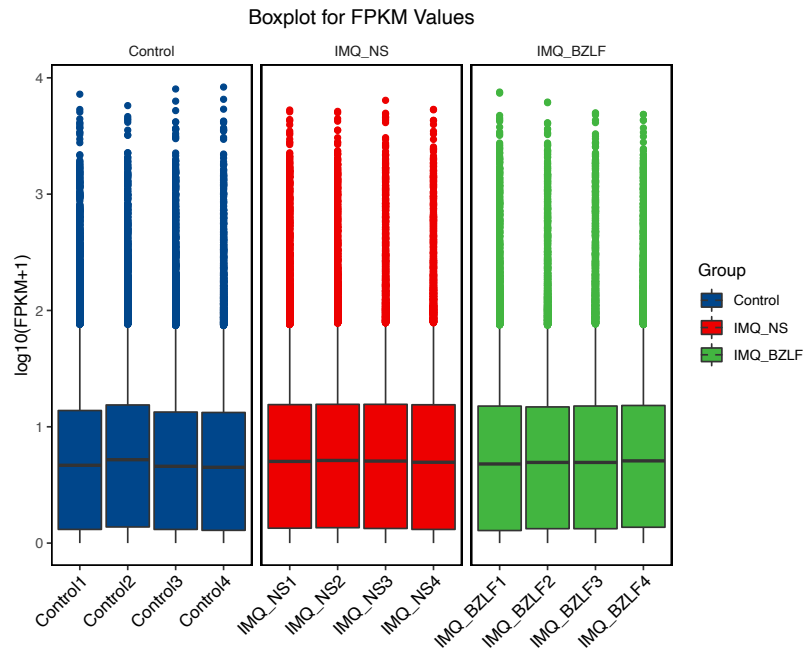**B**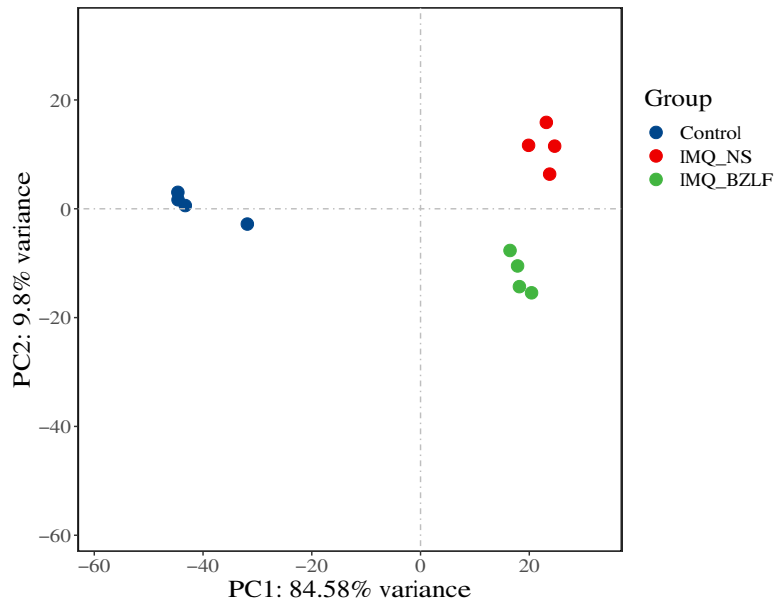

Supplement: Supplementary file 2 [file Image2.pdf]

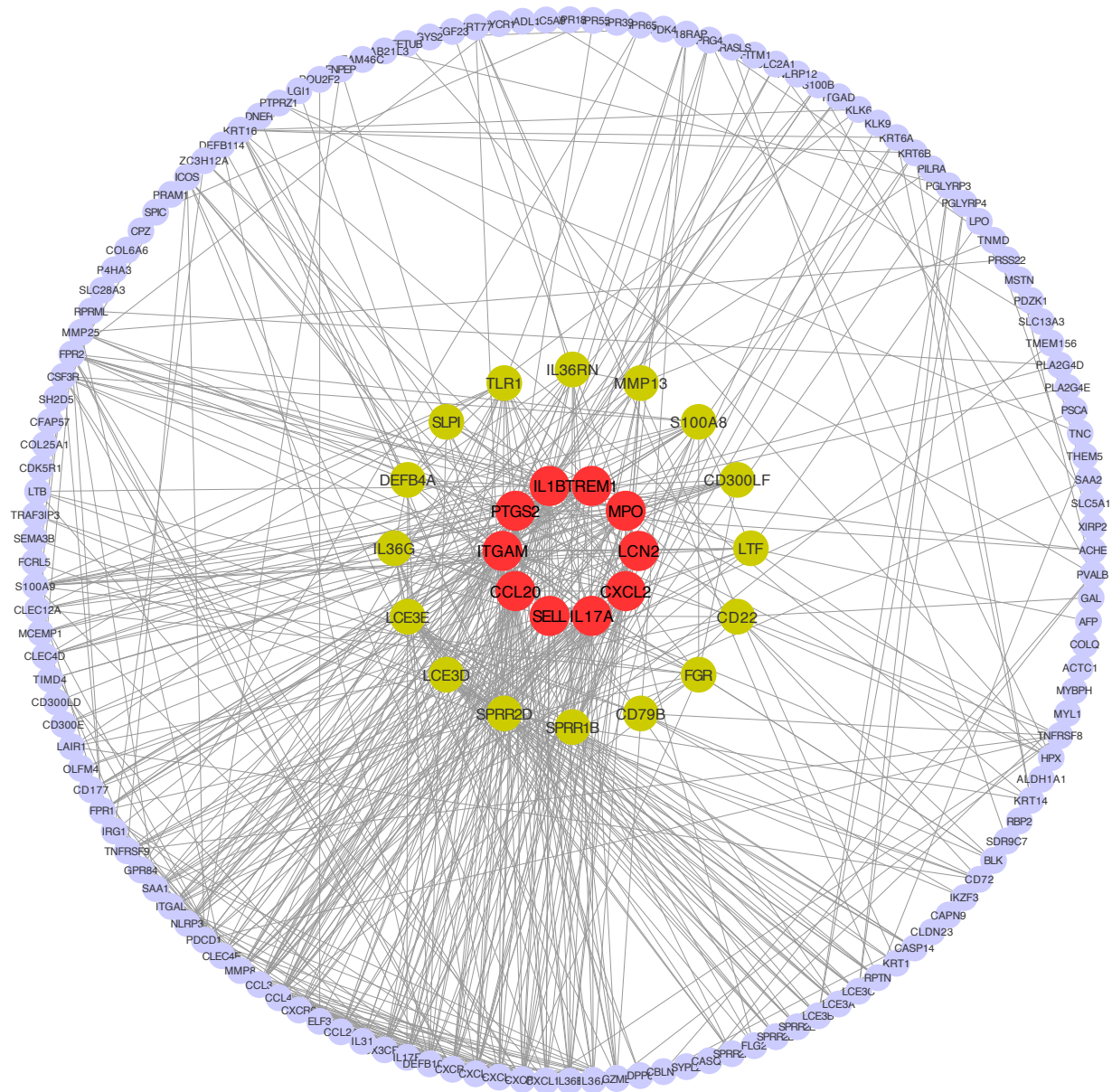

Supplement: Supplementary file 3 [file Image3.pdf]

**A**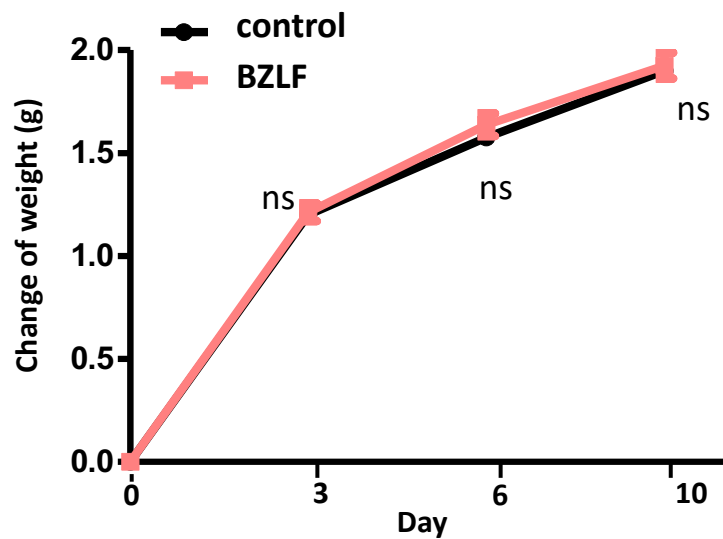**B**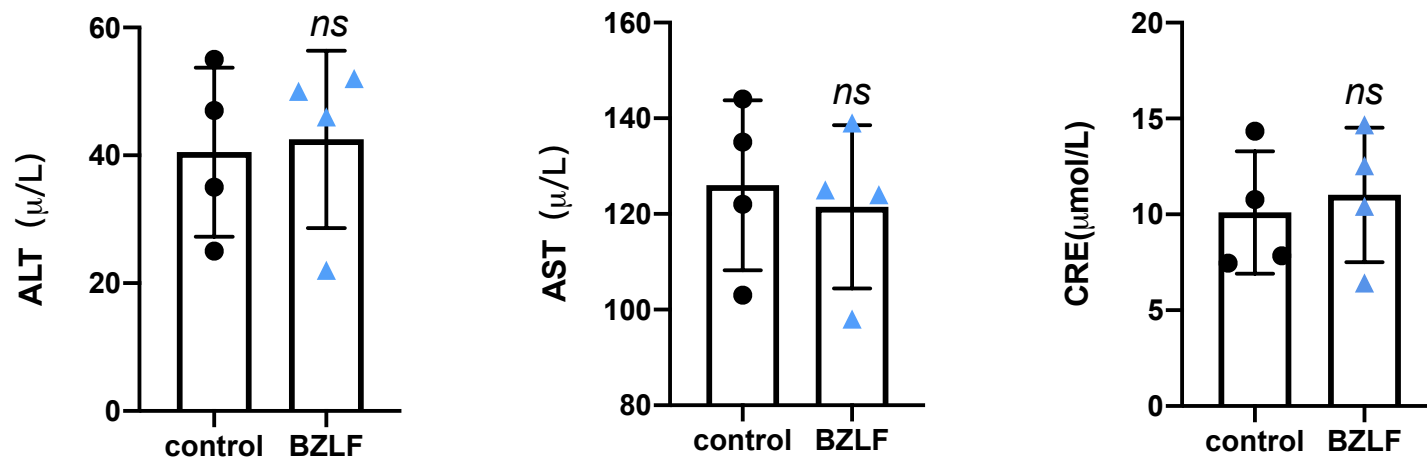

Supplement: Supplementary file 4 [file Image1.pdf]
